# A Double-Edged Sword: Breast Milk-Derived Maternal Antibodies and Infant Vaccine Responses: A Narrative Review

**DOI:** 10.3390/vaccines14070559

**Published:** 2026-06-25

**Authors:** Alexandra Mpakosi, Rafaela Anna Moutsopoulou, Stamatios Cholevas, Alexandra Lianou, Andriana Samata, Foteini Tziraki, Ioannis Vogiatzis, Vasileios Cholevas, Zoi Iliodromiti, Theodora Boutsikou, Nicoletta Iacovidou, Andreas G. Tsantes, Rozeta Sokou

**Affiliations:** 1 Department of Immunology, General Hospital of Nikaia “Agios Panteleimon”, 18454 Piraeus, Greece; samatadri@gmail.com (A.S.); paliotziraki@hotmail.com (F.T.); jvoyia.syr@gmail.com (I.V.); 2 Neonatal Department, National and Kapodistrian University of Athens, Aretaieio Hospital, 11528 Athens, Greece; moutsopoulourafaela@gmail.com (R.A.M.); ziliodromiti@yahoo.gr (Z.I.); theobtsk@gmail.com (T.B.); niciac58@gmail.com (N.I.); 3 School of Pharmacy, European University of Cyprus, Diogenes, Engomi, Nicosia 2404, Cyprus; stam17112004@gmail.com; 4 Neonatal Intensive Care Unit, “Agios Panteleimon” General Hospital of Nikea, 18454 Piraeus, Greece; alexlianou95@gmail.com; 5 School of Medicine, University of Bologna, 40126 Bologna, Italy; billcholevas34@gmail.com; 6 Department of Microbiology, Saint Savvas Oncology Hospital, 11522 Athens, Greece; andreas.tsantes@yahoo.com

**Keywords:** maternal antibodies, breast milk, infant vaccine responses, interference, maternal prenatal vaccination

## Abstract

Neonatal defense against pathogens relies on maternal antibodies transferred both through the placenta (IgG) and through breast milk (primarily secretory IgA). Maternal IgG antibodies are transferred across the placenta to the fetus mainly via the neonatal Fc receptor (FcRn), which is expressed at high levels in placental syncytiotrophoblasts, and results in the acquisition of maternal-fetal IgG. Transplacental transfer via the FcRn pathway can provide therapeutic proteins and protective antibodies following maternal vaccination. However, maternal IgG antibodies can bind to vaccine antigens such as measles, tetanus, and poliovirus, resulting in rapid clearance through FcgRIIB-mediated inhibition and inadequate B cell activation. In this way, they can inhibit de novo immune responses and significantly reduce vaccine response. On the other hand, the interference that breast milk-derived antibodies may have on vaccine-induced immunity is still largely unknown. Vaccination against influenza, pertussis, and COVID-19 during pregnancy or lactation has been shown to induce the production of protective, pathogen-specific, secretory IgA and IgG antibodies in breast milk. Conversely, studies found that breast milk-derived antibodies of vaccinated mothers reduced vaccine-induced immunity in breastfed infants by accelerating the clearance of vaccine antigen, resulting in reduced antigen availability and reduced plasma cell formation after vaccination. Additional factors in middle- and low-income countries, including environmental (increased microbiome diversity, environmental intestinal dysfunction, malnutrition, co-infections) and breastfeeding practices, may exacerbate the interference effect of maternal antibodies. Current evidence supports that breastfeeding is associated with a reduced immunological response exclusively to the rotavirus vaccine. However, the limited evidence base to date precludes definitive conclusions regarding the role of breast milk-derived antibodies in modulating vaccine-induced immunity. Nevertheless, the evidence suggests that although maternal antibodies may theoretically reduce vaccine immunogenicity, the overall protective benefits of breastfeeding outweigh any potential interference with vaccine responses.

## 1. Introduction

Newborns and infants have an immature immune system that makes them vulnerable to serious, potentially life-threatening infections. However, this gap in infant immunity is bridged by maternal antibodies that are passively transferred across the placenta and through human milk [1].

The only class of antibodies transferred across the placenta is maternal IgG [2]. This transfer begins at 13 weeks of gestation and continues throughout pregnancy, with the majority being transferred in the third trimester [3,4,5]. During transplacental transfer of maternal IgG, high concentrations are prevented by a mechanism based on saturation of the FcRn receptor. Factors that may influence transplacental IgG transfer include maternal total IgG, IgG subclass, gestational age, placental status, and the nature of the antigen [3,5,6]. Maternal IgG is then incorporated by endocytosis and binds to the neonatal receptor for the crystallizable (Fc) region of the IgG fragment (FcRn) on endosomes [4,7,8]. Maternal antibodies play a critical role in conferring passive immunity to the newborn; however, they may interfere with vaccine-induced immune responses, primarily by attenuating antibody production. This interference predominantly affects humoral immunity and does not appear to impair T-cell development, which is governed by the child’s own major histocompatibility complex-restricted genetic background and is not maternally transferred. Moreover, the maturation of T-cell–mediated immunity can proceed normally in the presence of maternal antibodies, with any modulatory effects being more pronounced on the magnitude of the response rather than on its initiation or occurrence [9].

As previously discussed, newborns are particularly susceptible to infections due to the immaturity of their immune system [10]. Their immature immune system is also partly responsible for their ineffective immune response to vaccine antigens [6,11]. Nevertheless, vaccination during pregnancy appears to be an effective strategy to protect not only the mother but also the developing fetus from infections, mainly through transplacental transfer of maternal antibodies. For example, during pregnancy, it has been an established practice to administer vaccines such as the inactivated influenza vaccine and the combined tetanus toxoid, reduced diphtheria toxoid, and acellular pertussis vaccine (Tdap) [6]. In addition, during the recent COVID-19 pandemic, vaccination during pregnancy appeared to provide protection against SARS-CoV-2, not only for the pregnant women themselves but also for their infants [12]. In recent years, maternal vaccination against respiratory syncytial virus (RSV) has been increasingly recommended as a preventive strategy to protect newborns through the transplacental transfer of maternal antibodies. It is particularly recommended for pregnant women without contraindications between 32 and 36 weeks of gestation who are expected to deliver during the RSV season (typically from September to January), especially when passive immunization with monoclonal antibodies is not planned for their infants [13,14]. Moreover, in special circumstances, safe vaccinations during pregnancy are considered to be those against hepatitis B, *Neisseria meningitidis* (Nm) with meningococcal polysaccharide vaccines, and polio with the inactivated virus vaccine (IPV) [15,16].

Breast milk, in addition to its critical role in supporting infant growth, development, and immune system maturation, can also play a crucial protective role against infectious diseases by providing a wide range of immune factors and bioactive components, including antibodies, cytokines, oligosaccharides and nucleic acids [17,18]. Of the immunoglobulins contained in human milk, IgA represents approximately 90% and plays a role in mucosal immunity, while IgM and IgG represent approximately 8% and 2%, respectively [19,20]. Vaccination during pregnancy or lactation also appears to offer protection not only to the mother but also to the infant from infections, mainly through the transfer of antibodies through human milk. For example vaccination against influenza, pertussis, and COVID-19 during pregnancy or lactation has been shown to induce the production of protective, pathogen-specific, secretory IgA and IgG antibodies in breast milk [21,22,23,24]. Furthermore, maternal vaccination with *Neisseria meningitidis* during pregnancy appeared to be safe for both mothers and infants and provided infants with increased levels of specific IgA in breast milk for 6 months [25]. Similarly, maternal immunization with pneumococcal polysaccharide vaccine in the third trimester of pregnancy appeared to be safe for both mothers and infants and resulted in high concentrations of IgA to all serotypes in breast milk in infants 2 months postpartum [26]. Conversely, other studies have shown that antibodies derived from the breast milk of vaccinated mothers may reduce vaccine-induced immunity in breastfed infants [27]. In fact, the interference that antibodies derived from breast milk may have on vaccine-induced immunity is still largely unknown. For example, a 2025 systematic review found that only 2 studies have addressed the disease in breastfed infants born to mothers who were vaccinated during pregnancy, and neither distinguished between the effects of antibodies via transplacental transfer, breast milk transfer, and naturally acquired antibodies [28]. In particular, the oral rotavirus vaccine may be affected in the intestinal mucosa by antibodies such as IgA, which are transferred to the infant via breast milk and which may reduce the efficacy of the vaccine. It is also possible that the interference mechanism caused by IgA differs from that caused by IgG due to differences in Fc characteristics. Furthermore, these differences are likely to be influenced by the main location in which these different antibodies are present, i.e., circulation compared to mucosal surfaces. Moreover, as already mentioned, breast milk, in addition to the main IgA antibodies it contains, also contains IgG antibodies, which can be transferred to the lamina propria and into the circulation [29,30,31]. Although breastfeeding and maternal vaccination during pregnancy are widely recognized as conferring substantial health benefits, their impact on vaccine responses in newborns is still not fully understood. Previous studies have not been able to clearly distinguish between antibodies originating from the mother and those produced by the infant in circulation, making it difficult to assess how maternal antibodies influence the infant’s own response to vaccination. In addition, the respective roles of antibodies transferred through the placenta and those delivered via breast milk remain difficult to separate when evaluating vaccine-induced immune responses in early life. This review summarizes current research on the mechanisms behind the interference phenomenon by which maternal antibodies from breast milk can affect vaccine-induced immunity in human infants.

## 2. Mechanisms of Interference

Several studies have shown that maternal IgG antibodies transferred to infants may inhibit vaccine effectiveness and antibody production possibly through B cell inhibition. In fact, the latter may be mediated by a cross-linking mechanism between the B cell receptor (BCR) and the Fcγ receptor IIB via a vaccine-antibody complex [9]. Another possible mechanism refers to the ability of maternal antibodies to induce rapid clearance of vaccine antigen by macrophages or to neutralize live attenuated vaccines, reducing the concentration of antigen available to stimulate the infant’s immature immune system. A third mechanism involves the ability of maternal antibodies to bind and mask epitopes on the vaccine antigen, resulting in their non-recognition by the infant’s B cell receptors [9]. However, no single mechanism has been established as universally responsible, as the magnitude of interference is influenced by both pathogen-specific factors and vaccine design characteristics. In addition to their ability to inhibit vaccine effectiveness, maternal antibodies also have the ability to inhibit the effectiveness of booster doses administered later in infancy. Furthermore, inhibition of vaccine effectiveness may result in lower long-term protection against pathogens later in childhood. On the other hand, it appears that high maternal antibody titers significantly limit antibody concentrations in infants after vaccination. For these reasons, and in order not to affect immune responses, many vaccination programs, such as measles-mumps-rubella (MMR) vaccination, are recommended to be carried out after maternal antibodies have declined, around 6–12 months of age [9,32,33].

**Figure 1:**
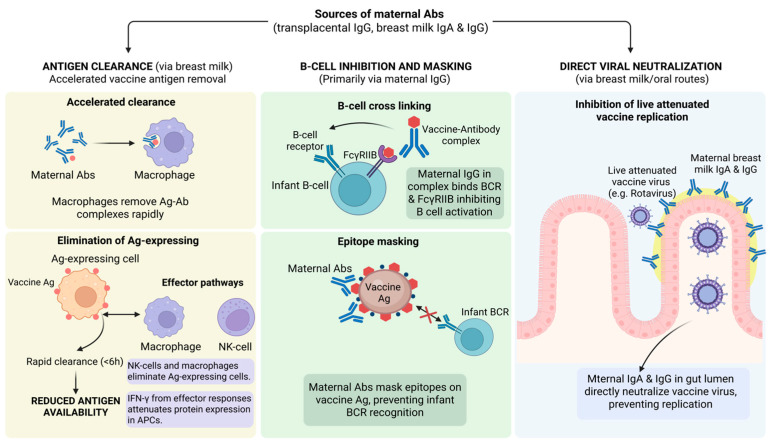
**Mechanisms of Interference**. Maternal antibodies transferred prenatally and through breast milk may attenuate infant vaccine responses by reducing vaccine antigen availability through B-cell inhibition, epitope masking, accelerated antigen clearance, and direct neutralization of live attenuated vaccine viruses. Among breast milk–mediated mechanisms, accelerated antigen clearance and direct viral neutralization appear most important, particularly for oral vaccines, as both limit antigen replication and immune stimulation in the infant, potentially reducing primary and booster vaccine immunogenicity. Created in BioRender. Lianou, A. (2026) https://www.biorender.com/. IFN-γ: Interferon gamma, Ag: Antigen, Ab: Antibody, NK-cell: Natural killer cell, APCs: Antigen-presenting cells, FcγRIIB: Fc receptor IIB for IgG, BCR: B-cell receptor.

Among the mechanisms mentioned above, two are the main ones by which antibodies derived from breast milk may interfere with vaccine-induced immunity: accelerated antigen clearance and direct virus neutralization. Both mechanisms reduce the availability and replication of the vaccine antigen, rendering it insufficient to stimulate the immature immune system of the breastfed infant, leading to reduced immune responses (Figure 1).

### 2.1. Antigen Clearance

Maternal antibodies transferred through breast milk may accelerate the clearance of vaccine antigens, resulting in a reduced concentration of antigens that is insufficient for immune system recognition and response. For example, Dangi T et al., used an experimental model and showed that breastfeeding by a mother vaccinated with mRNA-SARS-CoV-2 was associated with reduced vaccine-induced immunity in neonatal mice [27]. They also revealed that mice suckled by vaccinated mothers showed reduced plasma cell counts after vaccination, compared with mice suckled by unvaccinated mothers, and argued that passive antibody transfer through breastfeeding likely accelerated the clearance of vaccine Ag in suckling mice, reducing Ag availability. The authors report that their “luciferase reporter model” demonstrated rapid clearance of vaccine antigen within 6 h post-vaccination in pups that nursed from luciferase-immune dams. In this context, they suggest that immune cells such as NK cells and macrophages may eliminate antigen-expressing cells through antibody-dependent effector pathways. They further note that IFNγ produced during these antibody effector responses may attenuate protein expression in antigen-presenting cells without necessarily inducing cell death. The authors also emphasize that, although luciferase is primarily an intracellular protein, a fraction can be detected on the cell surface, making it accessible to antibody-mediated effector functions. Finally, they state that further studies are required to clarify the contribution of mechanisms such as dependent cell-mediated cytotoxicity and complement-mediated cytotoxicity in the clearance of vaccine antigen–expressing cells, and suggest that these findings may inform future vaccination strategies in infants breastfed by immune mothers [27].

### 2.2. Direct Viral Neutralization 

In the case of live attenuated vaccines administered orally, there is direct neutralization of the vaccine virus by breast milk antibodies, further preventing the replication of the virus that is necessary to trigger strong immune responses. For example, Kazimbaya KM et al. demonstrated that whole breast milk, purified IgG fractions, and purified IgA fractions all showed inhibitory activity against rotavirus vaccine strains [34]. They also hypothesized that mothers with high IgA and IgG titers in rotavirus endemic areas may inhibit virus replication in the infant’s intestine, possibly contributing to the failure of seroconversion in their infants [34]. Chandler TL et al. further investigated the underlying mechanisms by which high maternal antibody concentrations may be associated with poor seroconversion using a mouse model. They found that rotavirus vaccination does not induce seroconversion if maternal antibodies are present, as they can inhibit vaccine replication [35].

## 3. Impact on Different Vaccine Types

Vaccines can be classified based on their ability to replicate in the host (e.g., live attenuated versus inactivated vaccines) and on the technological platforms used in their production (messenger RNA (mRNA) vaccines, viral vector vaccines, subunit, recombinant and capsular polysaccharides or protein-conjugated capsular polysaccharides vaccines, toxoid vaccines) [36]. The differences in vaccine composition and mechanism of action may influence the magnitude, duration, and isotype distribution of the antibody response induced in breast milk, potentially affecting passive immunity transferred to the infant through breastfeeding [17].

Moreover, accumulating evidence suggests that different vaccine platforms induce distinct antibody responses in breast milk. Meningococcal polysaccharide, pneumococcal polysaccharide, and inactivated influenza vaccines have been associated with sustained increases in vaccine-specific IgA antibodies in breast milk for up to six months postpartum, while elevated IgG levels have been reported mainly during the first two postpartum months. These concentrations may be four- to five-fold higher than those detected in milk from unvaccinated mothers [21,25,26,37,38].

Conversely, maternal immunization with Tdap appears to induce a shorter-lived mucosal antibody response, with anti-pertussis IgA levels declining substantially within the first 2–6 weeks after delivery [22,39].

In addition, the inactivated influenza vaccine has been shown to elicit higher concentrations of influenza-specific IgA in breast milk compared with the live-attenuated influenza vaccine [40].

Furthermore, the potential influence of breast milk antibodies on the infant’s immune response to vaccination may depend not only on the quantity of maternally derived IgA and IgG transferred through breastfeeding, but also on the route of vaccine administration and the nature of the vaccine antigen. Oral vaccines interact directly with the intestinal mucosa, where maternal secretory IgA (SIgA) may neutralize vaccine antigens or attenuated microorganisms before they stimulate an effective immune response. This mechanism has been proposed for rotavirus vaccines, where transplacentally acquired IgG antibodies and immune factors present in breast milk may reduce vaccine immunogenicity in infants [41,42]. In contrast, parenterally administered vaccines, such as intramuscular vaccines, bypass the intestinal mucosal environment and are therefore less likely to be directly affected by breast milk antibodies [43,44].

Taken together, these findings suggest that the interaction between passive immunity conferred through breastfeeding and active immunization of the infant is likely to be vaccine-specific. Therefore, the extent to which breast milk antibodies modulate infant vaccine responses may vary according to both the route of administration and the immunological characteristics of the vaccine antigen, factors that should be considered when evaluating vaccine effectiveness during early infancy.

### 3.1. Oral Administered Vaccines

#### 3.1.1. Oral Rotavirus Vaccines

The most pronounced interference from breast milk antibodies is observed with rotavirus vaccines. In particular, maternal antibodies may interfere with oral rotavirus vaccination by accelerating viral clearance. As already mentioned, whole breast milk, purified IgA and purified IgG have inhibitory properties on rotavirus vaccine replication. In contrast, breast milk with reduced IgA and IgG has no inhibitory capacity [34,35]. In addition, early passive protection from the mother may reduce vaccine-induced immune responses in neonates when there are high titers of maternal rotavirus-specific antibodies from repeated exposures [45,46]. However, it has been previously shown that withholding breastfeeding around the time of rotavirus vaccination is unlikely to improve the vaccine immunogenicity [47,48]. Chandler et al. showed that the most likely mechanism of interference does not involve inhibitory signaling through FcγRIIB, but the formation of immune complexes between high titers of IgG and IgA transferred from the mother and vaccine particles, resulting in their clearance [35]. However, it seems that at very low doses of maternal antibodies, FcγRIIB may play a role by slightly reducing IgG responses. In contrast, inhibition of IgA production in newborns remains a fact that probably indicates the different immune responses to the vaccine depending on the isotype [46]. The authors explain that the FcγRIIB receptor, which is classically implicated in the inhibition of IgG-mediated immune responses, does not appear to be the primary mechanism responsible for maternal antibody interference with rotavirus vaccination in the context of natural infection. In FcγRIIB knockout (KO) mice, maternal antibody–mediated interference remained largely intact, suggesting that additional mechanisms are involved.

However, in passive transfer experiments involving very low levels of maternal antibodies, the absence of FcγRIIB was associated in some cases with the development of an IgG response in neonates, indicating a potential role of this receptor under specific conditions. The authors further report that previous studies in alternative models, such as those using sheep erythrocytes and measles vaccination, support a role for the Fc region of IgG in mediating suppression, as only IgG capable of engaging Fc receptors was able to induce inhibition [49,50].

Notably, the study also demonstrates that neonatal IgA responses are affected, indicating that maternal antibody interference is not restricted to IgG but may extend to multiple components of humoral immunity through distinct and partially independent mechanisms.

Overall, the authors conclude that maternal antibodies can suppress neonatal immune responses through redundant and multifactorial pathways, while emphasizing the need for further investigation into how early stages of the immune response, including IgM production and B cell differentiation, are regulated in the presence of maternal antibodies.

#### 3.1.2. Oral Polio Vaccine

Existing evidence regarding oral polio vaccine remains limited and inconclusive. Zaman S et al. determined specific antibodies against poliovirus type I in the breast milk of unvaccinated mothers before and seven years after the start of vaccination of their infants with oral polio vaccine [51]. They found that colostrum contained high levels of neutralizing antibodies that could potentially interfere with the oral poliovirus vaccine that is often administered on the day of birth [51]. On the other hand, oral polio vaccine appears to interfere with immune responses to rotavirus vaccines. The chances of interference actually increase when these vaccines are administered concomitantly [52]. A secondary, pooled analysis of Phase II and III trial data from 33 countries found that oral poliovirus vaccine administered to infants concurrently with monovalent rotavirus vaccine resulted in reduced IgA seroconversion against rotavirus, and that these interference effects occurred mainly after the second dose of rotavirus vaccine [53].

### 3.2. Parenterally Administered Vaccines

A meta-analysis of 32 studies involving 7630 infants found that preexisting maternal antibodies inhibited infant immune responses to 20 of 21 parenteral vaccine antigens. Maternal antibodies were predominantly transplacental IgG [54]. However, breast milk antibodies appeared to play a critical role in ongoing exposure. Among parenterally administered vaccines, inactivated poliovirus, diphtheria virus, pertactin (acellular component of pertussis), and tetanus were most affected by maternal antibody interference. Specifically, the twofold increase in maternal antibodies led to: 20–28% lower antibody concentrations after vaccination with the inactivated polio vaccine, 24% lower antibodies after vaccination for diphtheria, 22% lower antibodies after vaccination with pertactin, and 13% lower antibodies after vaccination for tetanus [54]. The interference effects of maternal antibodies were mainly evident from the first dose of vaccine. For this reason, when feasible, delaying the administration of the first vaccine dose by 2–5 weeks is recommended to allow maternal antibody levels to decline, thereby minimizing potential interference. However, for some vaccines, including acellular pertussis, inactivated poliomyelitis, and diphtheria, this interference continued even with booster doses at 12–24 months of age [54].

In secondary analyses reported by Björksjö et al., breastfed infants were compared with formula-fed infants as a combined group. All Swedish infants in the study received routine intramuscular vaccinations at 3, 5, and 12 months of age against diphtheria, tetanus, poliomyelitis, pertussis, hepatitis B, *Haemophilus influenzae* type b (Hib), and pneumococcal disease, using the Infanrix Hexa and Synflorix vaccines. Breastfed infants exhibited significantly lower anti-Hib IgG levels at 4 months and a reduced antibody response to all three vaccines at 6 and 12 months of age. In addition, a higher proportion of formula-fed infants achieved protective anti-Hib antibody concentrations at all time points, with a similar pattern observed for diphtheria, whereas tetanus antibody levels remained high and comparable between the two groups [55].

## 4. Age-Related Vulnerability to Reduced Vaccine Efficacy Due to Maternal Antibodies

Infant’s age at the first immunization appears to play a crucial role in the efficacy of vaccines due to interference from maternal antibodies. Indeed, vulnerability appears to decrease as infants grow older and their immune systems become more mature, while maternal antibodies decline. However, although the interference effect is most pronounced for the first dose of the vaccine, it may persist until booster vaccinations at 12–24 months of age [54]. Serum antibody concentrations were analyzed in more than 7600 infants from 17 countries, thereby addressing the limited statistical power that has constrained previous investigations of maternal antibody interference with vaccine immunogenicity. Their findings demonstrate that, for nearly all vaccine antigens, maternally derived (transplacentally transferred) antibodies suppress the immune response to the primary vaccine dose, and that this inhibitory effect is not entirely abrogated following booster immunizations. Moreover, they show that increased age at the time of initial vaccination is associated with enhanced immunogenicity, independent of maternal antibody titers. In contrast to earlier paradigms, the impact of maternal antibodies and age at priming is not confined to primary responses but may also extend to memory responses up to 12–24 months post-vaccination for multiple antigens, underscoring the critical importance of the quality of the initial antigenic encounter [56,57]. The authors further contextualize their findings within the existing literature, noting that previous studies have reported heterogeneous or partially discordant results depending on vaccine formulation and study design. These inconsistencies highlight the ongoing lack of consensus in the field. Finally, with respect to immunization schedules, the data indicate that extended intervals between vaccine doses may permit partial waning of maternally derived antibodies; however, interference is most pronounced at the level of the primary vaccine dose [54].

## 5. Maternal Antibody Decay Kinetics

Maternal antibody decay kinetics vary depending on antigenic specificity and IgG subclass. High concentrations of total IgG may lead to accelerated catabolism. Antibodies transferred across the placenta have a half-life ranging from 30 to 60 days [58]. On the contrary, breast milk antibodies may persist throughout breastfeeding, reaching up to 5–6 months after birth, although the duration varies depending on the specific antigen [9,59]. These maternal antibodies may transiently blunt infant vaccine responses by masking the antigen and accelerating vaccine clearance, but this interference is offset by the protection provided during early infancy [60,61].

### 5.1. Transplacental IgG Transfer

The half-life of IgG antibodies transferred from mother to infant depends on factors including antigen specificity, IgG subclass, and neonatal Fc receptor (FcRn) interaction. For example, half-lives for pertussis antigens are approximately 30–40 days, for tetanus 50 days, and for group B streptococcus 60 days [60]. For SARS-CoV-2 antibodies following maternal COVID-19 vaccination, the half-life in infants was approximately 42 days. However, protective antibodies were able to persist for 5–6 months in most infants whose mothers were vaccinated in the second or third trimester of pregnancy [62].

### 5.2. Breast Milk Antibodies

Breast milk contains both IgG and IgA antibodies, transferred via FcRn transport and local production by maternal B cells. Cassidy AG et al. investigated the persistence of milk-associated antibodies in mothers who had received a COVID-19 mRNA vaccine during pregnancy [62]. They found that median IgG and IgA titers remained positive in milk samples up to 5 to 6 months after birth. Furthermore, no difference in the rate of decay was observed between them, as both IgA and IgG titers declined significantly in milk between the 1 to 2-month and 5 to 6-month tests. However, booster vaccination during pregnancy appears to prolong the half-life of IgG in milk [62]. In contrast, antibody titers after pertussis vaccines remain high in milk samples only for about 4 weeks after vaccination, while after influenza vaccines, the duration of high antibody levels varies [28]. Therefore, following maternal vaccination during pregnancy or lactation, the persistence of interference effects of vaccine-induced breast milk antibodies usually varies depending on the vaccine.

## 6. Geographic Variation in Vaccine Efficacy

The impact of maternal antibodies from breast milk on vaccine efficacy may vary between countries, particularly between low- and middle-income countries and high-income countries, but the underlying reasons remain unclear [41]. This variation is most pronounced for oral rotavirus vaccines. In fact, clinical trials have shown that rotavirus vaccines have lower efficacy in low- and middle-income countries (40 to 60%) than in high-income countries (80 to 90%) [63,64,65,66,67,68]. This variation appears to be related to differences in maternal antibody levels, microbiome development, breastfeeding practices, breast milk components, and nutritional deficiencies.

### 6.1. Μaternal Antibody Levels

As mentioned above, high titers of maternal rotavirus-specific antibodies from repeated natural exposures, particularly in endemic settings, may reduce vaccine-induced immune responses in neonates [45,46]. The higher maternal antibody titers observed in low- and middle-income countries compared with high-income countries are likely attributable to greater exposure to natural pathogens. In this context, seasonal viral circulation may further contribute to fluctuations in maternal antibody titers, thereby influencing infant vaccine immunogenicity over time. Observational studies in such countries have revealed an inverse association between maternal antibody titers in serum and breast milk and infant immune responses to rotavirus vaccination [45,46].

### 6.2. Microbiome Development

A recent multicenter cohort study explored the effect of maternal antibodies on the immunogenicity of oral rotavirus vaccine in infants from Malawi, India, and the United Kingdom [69]. It was observed that in both India and Malawi, oral rotavirus vaccine shedding and seroconversion rates were significantly lower than in the United Kingdom, maternal rotavirus-specific antibodies in serum and breastmilk were negatively associated with oral rotavirus vaccine response, and that increased microbiome diversity was negatively associated with rotavirus vaccine immunogenicity. The authors concluded that high exposure to microbial agents during the first years of life may contribute to reduced vaccine efficacy. This study demonstrated an inhibitory effect of maternal antibodies on the immunogenicity of oral rotavirus vaccines in low- and middle-income countries. This effect may be partially attributable to reduced shedding of the oral rotavirus vaccine. The authors argue that the gut microbiota may represent one of the potential mechanisms influencing the response to the oral rotavirus vaccine, although it does not appear to be the sole or sufficient factor to fully explain the observed geographical differences in vaccine efficacy. Specifically, they report that increased microbial diversity in infants from low- and middle-income countries may be associated with a mucosal immune “hyporesponsive” state, which could limit vaccine immunogenicity. At the same time, certain bacterial taxa have been linked to modulation of immune memory, suggesting that the microbiota may influence early stages of immune activation [70,71,72,73]. However, the authors emphasize that these associations alone are insufficient to account for differences in oral rotavirus vaccine efficacy between populations, indicating that the gut microbiome likely acts in concert with other factors, such as maternal antibodies and environmental exposures [69].

### 6.3. Breastfeeding Practices

As mentioned above, maternal antibodies include placental IgG that is transferred during pregnancy and subsequently circulates in the infant mediating systemic interference, and breast milk IgA that has a direct effect on the infant’s intestine, inhibiting the proliferation of the vaccine virus in intestinal epithelial cells. There, maternal antibodies may form immune complexes with live attenuated vaccine viruses, leading to neutralization and accelerated clearance of the vaccine from the gastrointestinal tract [17]. This dual route of maternal antibody transfer may contribute to greater interference in settings with prolonged breastfeeding. As long as the child is breastfed, maternal IgA is continuously supplied through breast milk possibly providing a continuous source of interference. It has been shown that in areas where breastfeeding is common, such as in low- and middle-income countries, a significant neutralizing effect of breast milk against rotavirus vaccine is observed [74,75]. In some clinical trials, breastfeeding was withheld for 1 h (South Africa and Pakistan) or 30 min (India), before and after vaccination with monovalent rotavirus vaccine, and no differences in seroconversion rates were observed between infants who had withheld breastfeeding before vaccination and those who had not [47,48,76]. It has been hypothesized, however, that since the half-emptying time of the infant’s stomach ranges between 47 and 56 min, the vaccine may have had time to come into contact with breast milk in the stomach or intestines and therefore may have been neutralized before an immune response was produced [75,77]. In other studies, mainly from high-income countries, the effectiveness of the rotavirus vaccine was compared in breastfed and formula-fed infants and no statistically significant difference in the frequency of seroconversion was found between them [78,79,80,81,82]. However, a case–control study from Germany revealed that exclusively breastfed infants were less likely to seroconvert [83].

### 6.4. Breast Milk Components 

Vaccine efficacy may also be affected by other immune factors contained in breast milk, such as glycoproteins and mucins, which may inhibit rotavirus replication [75,84,85,86,87]. On the other hand, lactoferrin and lactadherin have the ability to reduce the infectivity of the vaccine strain of rotavirus. Higher levels of lactoferrin, lactadherin, IgA and neutralizing activity were found in breast milk samples from Indian and South African women than from American women [88]. In addition, a positive correlation was demonstrated between lactoferrin or IgA levels and neutralizing activity in breast milk samples from India and South Africa, but not in American samples. Furthermore, the inhibitory effect of lactoferrin was found to be dose- or species-dependent, while the inhibitory effect of lactadherin against rotavirus vaccines was found to be less effective [88]. In contrast, Mwila-Kazimbaya K et al., revealed an association between high concentrations of lactadherin in breast milk and lower seroconversion rate in Zambian infants after rotavirus vaccination [89]. According to the above, the synergistic effect of high levels of maternal antibodies combined with high levels of lactoferrin and lactadherin in breast milk from low- and middle-income countries may further enhance the interference effect. On the contrary, another study found no association between the innate immune factors lactoferrin, lactadherin, and tenascin-c and seroconversion of Nicaraguan infants to the oral rotavirus vaccine [90].

### 6.5. Nutritional Deficiencies

In addition to the effect of maternal antibodies from breast milk, nutritional deficiencies may also contribute to geographical variation in vaccine effectiveness, particularly reducing the effectiveness of oral vaccines in low- and middle-income countries and affecting infants’ immune responses. However, the heterogeneity of the data makes it difficult to draw firm conclusions and more studies are needed [91].

Vitamin A, zinc, iron, and protein-energy malnutrition are the main nutritional deficiencies that can reduce the effectiveness of vaccines, mainly in developing countries, such as those in sub-Saharan Africa and South Asia [92].

#### 6.5.1. Vitamin a Deficiency

Vitamin A deficiency affects approximately 29% of children worldwide, with rates reaching 44% in South Asia and 48% in sub-Saharan Africa [92,93]. However, vitamin A is vital for maintaining mucosal surfaces, enhancing mucosal immunity, T-cell differentiation, and antibody production [94]. Its deficiency has been shown to significantly reduce the effectiveness of oral vaccines (polio and rotavirus vaccines) and measles, with supplementation being known to increase seroconversion rates by up to 30% [92].

#### 6.5.2. Zinc Deficiency

Zinc is vital for thymus development, and lymphocyte proliferation, cellular immunity and T-cell function [95]. Unfortunately, zinc deficiency is widespread in middle- and low-income countries, including those in sub-Saharan Africa, where more than half of children appear to have serum zinc concentrations below the deficiency threshold [93]. Deficiencies contribute to poor antibody responses to vaccines against rotavirus, hepatitis B and others. Zinc supplementation enhances responses to oral vaccines by approximately 20% [92].

#### 6.5.3. Iron Deficiency

Iron deficiency at the time of vaccination may also contribute to reduced vaccine response. As demonstrated in a previous study, hemoglobin levels were the strongest positive predictor of IgG responses to diphtheria and pertussis in Kenyan infants, and iron supplementation at the time of vaccination resulted in increased anti-measles IgG, seroconversion, and antibody avidity [96].

#### 6.5.4. Protein-Energy Malnutrition

Protein-energy malnutrition causes damage to lymphoid tissues (thymus and spleen), contributing to lower antibody responses to multiple vaccinations, such as measles, tetanus, and BCG vaccines [92].

## 7. Environmental Enteric Dysfunction (EED)

Environmental Enteric Dysfunction (EED) is characterized by alterations in intestinal structure and function and is commonly evaluated using biomarkers that reflect four key pathological processes: impaired intestinal barrier integrity, epithelial injury and regeneration, intestinal inflammation, and microbial translocation associated with immune activation [97,98,99]. These pathological domains have been proposed as potential contributors to the reduced effectiveness of oral vaccines, since damage to the intestinal mucosa may interfere with antigen uptake and mucosal immune responses, while chronic inflammation can modify immune regulation within the gut. However, evidence concerning the contribution of EED to oral vaccine performance remains inconclusive and somewhat controversial [100]. In low- and middle-income countries, these deficiencies often coexist with environmental enteropathy, in which frequent infections and chronic intestinal inflammation, combined with poor hygiene, affect intestinal absorption, linking malnutrition to vaccine inhibition [101,102]. Furthermore, during EED, local CD4+ T cell responses in the small intestine are inhibited, while systemic responses remain intact, resulting in a direct impact on the efficacy of oral vaccines [103]. For example, biomarkers of EED, such as intestinal fatty acid binding protein (I-FABP) and soluble CD14, have been associated with reduced seroconversion to rotavirus and other vaccines [104]. Nevertheless, the relationship between EED and the effectiveness of oral vaccines remains inconclusive. In a large birth cohort study conducted in Zimbabwe, no evidence was found that EED biomarkers adversely affected immune responses to the oral rotavirus vaccine, suggesting that interventions targeting EED alone may be insufficient to improve oral vaccine performance [105]. In contrast, Naylor et al. reported that environmental enteropathy was associated with Rotarix^®^ vaccine failure, reduced immunogenicity of the oral polio vaccine and an increased risk of malnutrition [106]. The authors proposed that chronic exposure to enteric pathogens, even in the absence of overt diarrheal disease, may induce persistent intestinal inflammation and immune activation, thereby compromising both vaccine responses and nutritional outcomes. Taken together, these findings highlight the complexity of the relationship between EED and oral vaccine efficacy. Although current evidence remains inconsistent, EED, intestinal and systemic inflammation, and environmental and sanitation-related factors appear to play important roles and warrant further investigation, particularly in low- and middle-income countries where oral vaccine underperformance is most frequently observed.

## 8. Specific Populations

Maternal antibody interference affects all infants to some extent. However, several specific populations face a higher risk of reduced vaccine response due to prolonged or elevated levels of maternal antibodies.

### 8.1. Preterm Infants

In premature infants, especially those born before 28 weeks of gestation, transplacental transfer of maternal antibodies is reduced compared to full-term infants, leading to lower absolute levels of these antibodies at birth. However, the half-life of maternal antibodies in premature infants is longer compared to full-term infants, leading to a prolongation of the window of vaccine interference [107]. Therefore, the immune responses of premature infants to vaccines will depend on the balance between lower initial levels of maternal antibodies that reduce interference on the one hand, and the prolonged persistence of maternal antibodies that may prolong interference on the other.

### 8.2. HIV-Exposed Uninfected (HEU) Infants

In infants born to HIV-infected mothers, the transfer of maternal antibodies is affected by maternal immune dysfunction. Thus, these infants present reduced transplacental transfer of vaccine-specific antibodies (Hib, pertussis, tetanus), altered maternal antibody profiles, and lower levels of cord blood antibodies against vaccine-preventable diseases compared with infants not exposed to HIV [108]. Surprisingly, these infants experience enhanced vaccine responses due to lower baseline maternal antibodies and therefore reduced maternal antibody interference. Thus, they exhibit similar or even enhanced antibody responses to pertussis, pneumococcus, and measles compared to infants not exposed to HIV [108,109].

### 8.3. Infants in Settings with High Maternal Antibody Levels

This includes infants of vaccinated mothers in high-coverage settings as well as infants of mothers in environments with high rates of natural infection that result in elevated maternal antibody titers. In these areas, delaying the first vaccination of infants by 2.2–5.0 weeks has been suggested to compensate for the interference of increased maternal antibodies [54].

## 9. Potential Mitigation Strategies

### 9.1. Withholding Breastfeeding

As mentioned above, in studies from countries such as South Africa, Pakistan and India, breastfeeding was interrupted for 30 min (India) and 1 h (South Africa and Pakistan) before and after oral vaccine administration, but without improvement in immunogenicity [47,48,76]. However, any potential benefit to vaccine outcome that may arise from an extended time window would have to be weighed against nutritional detriment and is therefore not likely to be practical.

### 9.2. Delayed Vaccination

Delaying the first vaccination contributes to both reduced interference from maternal antibodies and increased infant age and subsequent maturation of the immune system. For example, delaying the first dose of pertussis vaccine by 2.2–5.0 weeks can offset a 2- to 5-fold increase in maternal pertussis antibodies from prenatal vaccination. Similarly, vaccination delays of 2.6 to 5.9 weeks for diphtheria and 1.7 to 3.9 weeks for tetanus are recommended [54]. Additionally, a randomized trial in the Netherlands showed that a delayed vaccination schedule, including the first dose at 3 months instead of 6–8 weeks, administered to infants of mothers who had received prenatal Tdap vaccination, led to a reduction in maternal antibody interference without compromising infant protection [110]. Indeed, ensuring the protection of the infant from diseases during the vulnerable early infancy period must be non-negotiable and, therefore, before implementing any schedule, the reduced immunogenicity of the vaccine must be balanced against the risk of infant diseases. One solution would be high coverage of maternal prenatal vaccination to ensure protection of the infant in the delay period, as maternal antibodies provide immediate protection for most infants in the first months of life [54].

### 9.3. Wider Dose Spacing

As already mentioned, the interference is mainly mediated through the first dose. Schedules with longer dosing intervals allow for the decay of maternal antibodies between doses. However, some degree of inhibition is maintained regardless of the dosing intervals, suggesting that the interference is mainly through the effect on the response to the first dose [54]. Nevertheless, it appears that the interference and subsequent blunting of the infant’s antibody responses are transient and that antibody levels increase as the doses progress [111]. Wider dose spacing combined with additional booster doses may also be a promising approach. A recent systematic review found moderate to high levels of evidence that vaccine immune responses were higher when the additional rotavirus vaccine dose was administered as a booster dose in the second six months of life rather than as an extended primary course [112].

### 9.4. Vaccine Antigen Content and Dose

Some studies suggest that the antigen content and dose of infant vaccines may overcome the interference. For example, acellular pertussis vaccines exhibit less interference than whole cell vaccines in infants with high maternal antibody titers, possibly due to higher antigen load in acellular formulations [60]. Furthermore, in the case of conjugate vaccines, the carrier protein appears to play an important role. For example, infants with high maternal tetanus antibodies show reduced responses to *Haemophilus influenzae* type b vaccines conjugated with tetanus toxoid, but not to those conjugated with CRM197 [60].

### 9.5. Alternative Delivery Routes

Breast milk antibodies provide direct mucosal interference at the site of vaccine replication. Thus, the potential for parenteral vaccines to completely bypass the intestinal mucosal barrier is being investigated without clear conclusions yet [29]. Breast milk-derived antibodies may influence the immunogenicity of oral vaccines primarily through local mechanisms within the intestinal mucosa, where limited replication of the attenuated virus is required to elicit an adequate immune response. In this context, secretory IgA and, to a lesser extent, IgG present in breast milk may bind to vaccine–derived viral particles, resulting in neutralization or inhibition of their attachment to and entry into intestinal epithelial cells [113,114].

This antibody–mediated activity may facilitate enhanced clearance of oral vaccine antigen from the intestinal surface, thereby reducing local antigen expression and limiting antigen availability for subsequent immune activation. Consequently, the induction of robust mucosal and systemic immune responses may be attenuated [115,116].

**Figure 2:**
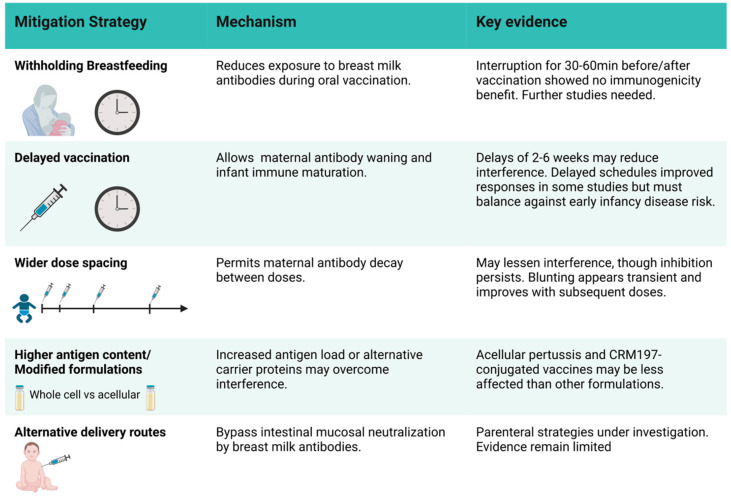
**Potential mitigation strategies summarized.** Created in BioRender. Lianou, A. (2026) https://www.biorender.com/.

Based on these proposed mechanisms, alternative vaccine delivery strategies, such as parenteral administration, are currently being investigated to circumvent the intestinal mucosal barrier and avoid interference from maternal antibodies. The main challenge here is inducing protective mucosal responses from a parenteral vaccine route. However, the available evidence regarding the efficacy of this approach remains limited (Figure 2).

## 10. Policy Implications

In settings with well-established prenatal immunization programs and high vaccination coverage, delaying the first vaccination could increase the infant’s response to the vaccine while maintaining protection through maternal antibodies during early infancy. However, as mentioned above, decisions about vaccination strategies must balance the immediate protection provided by maternal vaccination in early infancy against the increased risk of disease later in infancy due to reduced immunogenicity, taking into account local disease epidemiology and prenatal program coverage. Thus, in settings with well-established maternal vaccination programs that provide high coverage, modest delays (for example, delaying the start of the first dose by two additional weeks) appear to lead to reduced interference from maternal antibodies while maintaining the protection they provide during early infancy [54]. On the contrary, in settings without strong maternal vaccination programs or with a high disease burden, policy decisions should focus on the timely protection of infants during the early infancy. Indeed, delayed immunization programs in vulnerable age groups pose a significant risk of serious morbidity and mortality, particularly for infants under 3 months of age who face the highest risk from vaccine-preventable diseases [111].

## 11. Balance of Risks and Benefits

Maternal antibodies in breast milk provide significant protective benefits to infants, including passive immunity against infections, microbiome shaping, and immune system development, which far outweigh the concern of transient blunting of vaccine response [117,118].

### 11.1. Benefits of Breast Milk Antibodies

As is known, breast milk secretory IgA antibodies (SIgA) can inhibit the adhesion of pathogens to intestinal and respiratory mucosal surfaces, neutralize toxins, and prevent invasion of epithelial barriers [60]. Furthermore, SIgA shapes the infant’s gut microbiota during the critical first years of life through selective pressure, limiting certain bacterial species, and promoting beneficial symbionts [117]. The protective benefits of IgA antibodies in breast milk are particularly important in low- and middle-income countries where intestinal inflammation and malnutrition are common, in maternal diseases that affect transplacental IgG transport but do not affect IgA transport through breast milk, such as HIV infection, and in premature infants in whom it is transferred in increased amounts in colostrum [60]. On the other hand, IgG in breast milk provides additional protection through antibody-dependent neutralization and cellular cytotoxicity [60]. Breast milk antibodies also contribute to the development of the infant’s immune system through anti-idiotypic antibodies, cytokines, and immune cell transfer. In addition, breastfeeding may provide enhanced vaccine responses and long-term protection against immune diseases such as allergies and celiac disease [119].

### 11.2. Potential Risks of Breast Milk Antibodies

As already mentioned, the primary concern is that maternal antibodies may blunt the vaccine response by accelerating the clearance of vaccine antigen in the neonate. However, although maternal antibodies—transferred transplacentally and through breast milk—may inhibit infant antibody production after vaccination, T-cell development and cellular immune response remain largely intact, contributing to good responses to booster doses [9,120]. Therefore, these effects are transient and are offset by the protection provided by maternal antibodies during early infancy [61]. The secondary concern that breast milk IgA could interfere with oral vaccine responses has not been consistently demonstrated and remains a matter of investigation [60].

## 12. Conclusions

In conclusion, it is well established that breast milk contains vaccine-induced antibodies following maternal immunization during pregnancy or lactation. These breast milk-derived antibodies may transiently reduce the immunogenicity of infant vaccines, particularly orally administered vaccines, by accelerating antigen neutralization in the gastrointestinal tract, thereby potentially reducing infant antibody responses. Importantly, this effect appears to be most relevant for oral live vaccines, whereas evidence for clinically meaningful interference with parenteral vaccine responses remains limited and inconsistent. However, current evidence suggests that the transient blunting of infant humoral responses by maternal antibodies is offset by the protection provided by them during early infancy, when infants are most vulnerable. In addition, the clinical consequences of reduced vaccine efficacy in breastfed infants are outweighed by the protective benefits of breastfeeding. Importantly, breastfeeding remains a fundamental public health intervention, providing broad and well-established benefits including protection against infectious morbidity and mortality, support of immune system maturation, and long-term health advantages. Overall, current evidence supports the continued promotion of breastfeeding in conjunction with maternal immunization strategies, without evidence of clinically meaningful compromise in vaccine effectiveness. Future research should prioritize quantifying the contribution of breast milk-derived antibodies across different vaccine platforms particularly distinguishing oral from injectable vaccines, while also elucidating their interactions with cellular and mucosal immune responses and their impact on long-term protective efficacy. Furthermore, rigorously designed prospective studies with clinically relevant endpoints are required to establish the true clinical significance of early immune modulation and to inform the optimization of maternal and infant immunization strategies.

## Data Availability

Data are contained within the article.
